# New Core-Shell Nanostructures for FRET Studies: Synthesis, Characterization, and Quantitative Analysis

**DOI:** 10.3390/ijms23063182

**Published:** 2022-03-16

**Authors:** Anna Synak, Elżbieta Adamska, Leszek Kułak, Beata Grobelna, Paweł Niedziałkowski, Piotr Bojarski

**Affiliations:** 1 Faculty of Mathematics, Physics and Informatics, University of Gdansk, Wita Stwosza 57, 80-308 Gdansk, Poland; piotr.bojarski@ug.edu.pl; 2 Faculty of Chemistry, University of Gdansk, Wita Stwosza 63, 80-308 Gdansk, Poland; elzbieta.adamska@ug.edu.pl (E.A.); beata.grobelna@ug.edu.pl (B.G.); pawel.niedzialkowski@ug.edu.pl (P.N.); 3 Faculty of Technical Physics and Applied Mathematics, Gdansk University of Technology, Narutowicza 11/12, 80-233 Gdansk, Poland; kulak@mif.pg.gda.pl

**Keywords:** core-shell nanostructures, TiO_2_@SiO_2_, FRET, luminescent materials

## Abstract

This work describes the synthesis and characterization of new core-shell material designed for Förster resonance energy transfer (FRET) studies. Synthesis, structural and optical properties of core-shell nanostructures with a large number of two kinds of fluorophores bound to the shell are presented. As fluorophores, strongly fluorescent rhodamine 101 and rhodamine 110 chloride were selected. The dyes exhibit significant spectral overlap between acceptor absorption and donor emission spectra, which enables effective FRET. Core-shell nanoparticles strongly differing in the ratio of donors to acceptor numbers were prepared. This leads to two different interesting cases: typical single-step FRET or multistep energy migration preceding FRET. The single-step FRET model that was designed and presented by some of us recently for core-shell nanoparticles is herein experimentally verified. Very good agreement between the analytical expression for donor fluorescence intensity decay and experimental data was obtained, which confirmed the correctness of the model. Multistep energy migration between donors preceding the final transfer to the acceptor can also be successfully described. In this case, however, experimental data are compared with the results of Monte Carlo simulations, as there is no respective analytical expression. Excellent agreement in this more general case evidences the usefulness of this numerical method in the design and prediction of the properties of the synthesized core-shell nanoparticles labelled with multiple and chemically different fluorophores.

## 1. Introduction

Core-shell type nanoparticles are hybrid structures, made of at least two compounds that differ in chemical composition or phase structure. Their unique properties allow a wide range of potential applications. Typically, they show low toxicity, exhibit physical and chemical stability, and the required fluorophores can be easily introduced into discrete layers of the core. Core-shell nanoparticles usually consist of an inorganic nanocore, the properties of which can be tailored according to specific needs, and one or more (organic or inorganic) layers forming the shell. The desired features of such core-shell particles can be obtained by an appropriate combination of individual materials of core and shell as well as by adjusting the core radius to shell thickness ratio. The functionalized core-shell nanostructures with silica as a shell are used for application in many branches of nanotechnology for sensors design [1], such as a safe sunscreen [2], or as a catalyst in biodiesel production [3]. Various fluorophores can be introduced into or attached to core-shell particles to increase their solubility or photostability [4,5,6,7,8]. Suitably designed silica nanostructures form a representative example here as they not only limit photobleaching but also ensure good solubility of dyes in water and other important solvents (ethanol) [9,10]. Core-shell nanocomposites, which are labelled with numerous fluorophores, can, in the nanoscale, act similarly to antenna systems. Due to the small distance between fluorophores, they can efficiently collect and further exchange excitation energy. As a result, a broadband fluorescent system can be obtained in the nanoscale with the emission significantly shifted to the red. Moreover, the whole acceptor set can, under specific conditions, amplify the nonradiative energy transfer, increasing its sensitivity as a spectroscopic nano-ruler even to distances significantly exceeding 10 nm [11,12]. Core-shell nanostructures seem to be promising candidates to develop such desired materials for amplified energy transfer.

Förster resonance energy transfer (FRET) is the radiationless transfer of excitation energy occurring between the singlet excited state of the donor (D) and the ground state of the acceptor (A) through dipole-dipole interaction [13]. This process plays a very important role in prolific areas of science and technology, including the study of protein-protein interactions, the intramolecular distance between selected sites of biospecies, the study and design of optical properties of functional materials, nanostructures, molecular mono- and multilayers, interfaces, aggregates, plasmonically enhanced broadband emission or some ordered systems [14,15,16,17,18,19,20,21,22,23].

The efficiency of Förster resonance energy transfer (FRET) depends on the number of fluorophores, intermolecular distance, the relative orientation of transition dipole moments of interacting species, and restricted motions of fluorophores attached to the shell. Thus FRET can reflect the structural properties of the core-shell systems and yield important information on these nanostructures from the fluorescence decay curves. In this paper, we present an antenna like system for which the donor and acceptor of the electronic excitation energy were covalently bound (in different molar ratios) to the modified TiO_2_@SiO_2_ core-shell type nanostructures. We focus on two physically extremely different cases: (1) at a low ratio of donor to acceptor number (*N_D_*/*N_A_* << 1), energy transfer occurs in a single step from the excited donor to the vicinal unexcited acceptor, and (2) for the opposite case (*N_D_*/*N_A_* >> 1), energy migration occurs many times between donors preceding the energy transfer to acceptors.

Recently, we presented the theoretical model of FRET valid for single step energy transfers taking into account the size distribution of core-shell nanoparticles [24]. The coherence of the model was positively tested by the Monte-Carlo method, but until now, no experimental verification has been proposed. We would like to fill this gap by presenting and analysing donor fluorescence intensity decay in the presence of energy transfer. Simultaneously, we would like to find out the effect of energy migration followed by energy transfer to acceptors on the donor fluorescence intensity decay in a core-shell nanostructure. Although for this more general situation no theoretical model for core-shell nanoparticles has been formulated so far, the numerical analysis of experimental data can be performed by the powerful Monte-Carlo simulation method.

It is noteworthy that both described cases are important not only to verify respective models. Energy transfer is a very sensitive effect concerning the number of acceptors, and therefore it should be an effective tool to estimate the mean number of fluorophores bound to core-shell nanoparticles. It can also provide global information on the size distribution of synthesized nanoparticles. Besides the mentioned important physical information, such donor-acceptor antenna like systems can form a solid line for FRET-based communication at the nanoscale and act as a detector of selected vicinal properties of the medium like local organization, polarity, or viscosity. This also seems to be an interesting way to gain spectrally broadband intensive sources of light in the nano- or sub-microscale.

## 2. Results and Discussion

### 2.1. Synthesis and Analysis of TiO_2_@SiO_2_-(CH_2_)_3_-NH-D/A Nanocomposite

**Scheme 1:**
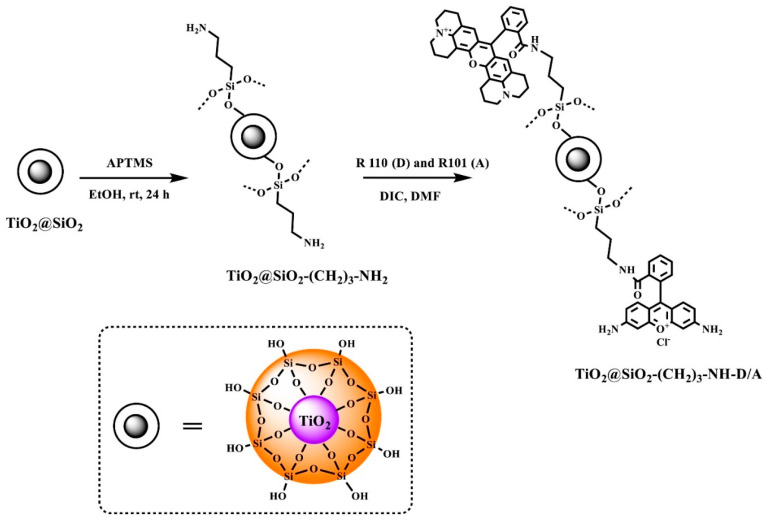
Synthesis of TiO_2_@SiO_2_-(CH_2_)_3_-NH-D/A.

The preparation of the TiO_2_@SiO_2_-(CH_2_)_3_-NH-D/A nanocomposite was achieved in several steps as illustrated in Scheme 1.

The TiO_2_@SiO_2_ core-shell nanostructures were synthesized using the Ströber method, which involves a sol-gel process. As a result of hydrolysis and condensation reactions, a silica layer is formed on the TiO_2_ surface. The faster hydrolysis reaction and slower condensation reaction of a silica precursor, i.e., tetraethoxysilane (TEOS) is possible thanks to the addition of ammonia as a catalyst. The addition of ammonia favors the obtaining of the final product of a silica shell with high porosity and surface area [25].

The modification of a core-shell nanostructure surface with amino groups (TiO_2_@SiO_2_-(CH_2_)_3_-NH_2_) was made using the procedure described earlier by us [26]. The nanostructures were obtained using 3-(aminopropyl)trimethoxysilane (APTMS) in the presence of toluene at its boiling point. The amino-functionalized nanostructures were used to attach fluorophores. In our case rhodamine 101 and rhodamine 110 chloride were covalently attached to the TiO_2_@SiO_2_-(CH_2_)_3_-NH_2_ structures. The synthesis was carried out with the *N*,*N*′-diisopropylcarbodiimide used in the peptide chemistry and an organic solvent, i.e., *N*,*N*-dimethylformamide.

The determination of the concentration of rhodamines attached to TiO_2_@SiO_2_-(CH_2_)_3_-NH_2_ was made as described in [27]. In the first step, the number of the Fmoc groups present on calcined TiO_2_@SiO_2_-(CH_2_)_3_-NH_2_ surface was estimated in the range 10 to 12 µmol/g. On this basis, the rhodamine concentration was calculated as 2 × 10^−4^ M. However, in the case of non-calcined TiO_2_@SiO_2_-(CH_2_)_3_-NH_2_ material, the amount of Fmoc groups is 6 to 9 µmol/g [27]. These calculations show that the calcination process leads to the increase of the number of active sites in the case of calcined TiO_2_@SiO_2_-(CH_2_)_3_-NH_2_ material. The reason for this difference is the increase of the silica surface of the calcined TiO_2_@SiO_2_ nanostructure.

**Figure 1:**
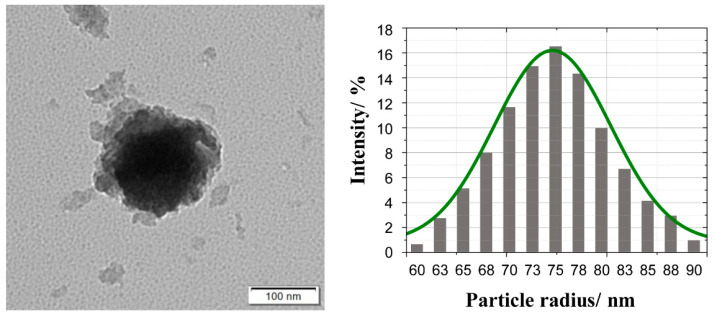
TEM image and diagram of the particle diameter of TiO_2_@SiO_2_-(CH_2_)_3_-NH-D/A nanoparticle. The solid green line corresponds to the Gaussian distribution with the mean value 〈R〉 = 74.58 nm and variance σ = 5.82 nm.

In order to determine the morphology of the designed nanocomposite, transmission electron microscopy (TEM) was used. Figure 1 shows a typical image of TiO_2_@SiO_2_-(CH_2_)_3_-NH-D/A nanoparticle upon real experimental conditions. As our previous research on this type of structure shows [27], in the TEM image, the dark part corresponds to the core, while the light gray part represents the shell. The shape of the nanostructures can be roughly approximated as a spherical one. Please note that the energy transfer process appears at a single sphere isolated by an appropriate distance from other core-shell nanoparticles. In our previous paper [27] we have shown that single TiO_2_ nanoparticles exhibit the tendency to agglomeration. However, the covering of TiO_2_ nanoparticles by silica shell and further their functionalization with amino groups derived from APTMS prevents the aggregation process.

Moreover, Figure 1 shows the statistical distribution of the obtained core-shell nanoparticles radius. Almost symmetric distribution can be seen with the maximum number of the obtained nanoparticles located around particle radius R = 75 nm with the halfwidth around 13 nm.

**Figure 2:**
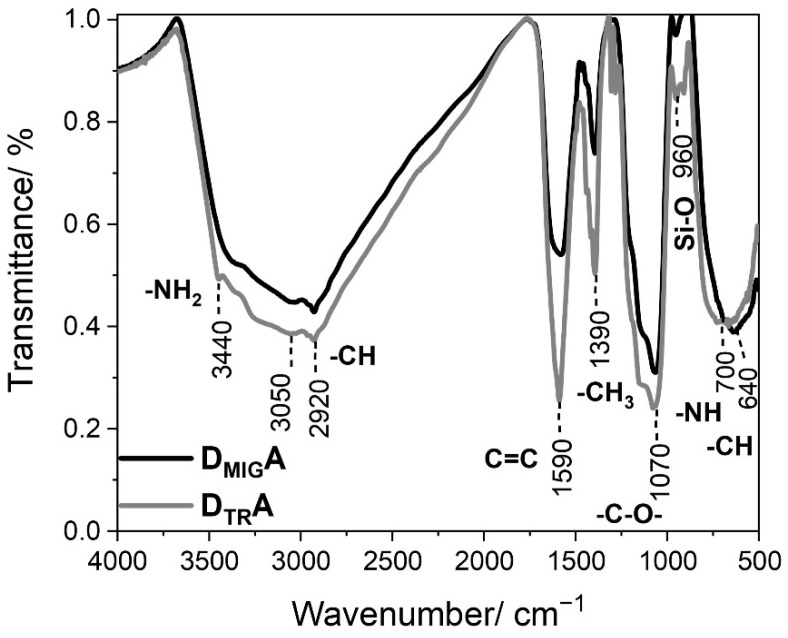
Fourier transform infrared spectra of TiO_2_@SiO_2_-(CH_2_)_3_-NH-R101/110 with a different ratio of the donor (D—rhodamine 110 chloride) to acceptor (A—rhodamine 101).

Fourier-transform infrared spectroscopy (FT-IR) was used to characterize the surface of the synthesized nanocomposite with the attached fluorophores. It enables the identification of characteristic functional groups present in each material (Figure 2).

The broad band ranging from 3000 to 3500 cm^−1^ is related to OH stretching vibrations, including those of water. Also, in this range, the peak with the maximum located at 3440 cm^−1^ reflects the stretching vibration of aminopropyl segments participating in hydrogen bonding with the silanol group in the nanocomposite [28]. However, the peaks at 3050 and 2920 cm^−1^ are associated with the stretching vibration of -CH aromatic groups in rhodamines, and -CH alkyl groups bound to the surface of silica, respectively [27,29]. Moreover, peaks located at 1590 cm^−1^ can be assigned to the C=C bending vibration, characteristic in aromatic molecules. Another intense band located at 1390 cm^−1^ was identified as bending in the -CH_3_ group [30]. However, the peak at 1070 cm^−1^ corresponds to the C-O stretching vibration of the ether [28,31]. Additionally, a weak band located at 960 cm^−1^ can be ascribed to the bending vibration of the Si-O group, and a more intense band located at 700 cm^−1^ can be ascribed to -NH bending vibration [26,32]. Moreover, the absorption band located at 640 cm^−1^ can be assigned to the C-H bending vibration in aromatic structures [32].

The zeta potential of the obtained core-shell nanostructures is an important parameter which provides a lot of information. Above all, it can be used in the measurement of the stability of the colloidal system. For future potential applications, it may also be considered as a means of predicting the in vivo behavior of nanomedicines. It is known that a suspended colloidal system is unstable if its zeta potential magnitude lies between +25 to −25 mV, and is stable for values greater than +25 mV or smaller than −25 mV [33].

**Figure 3:**
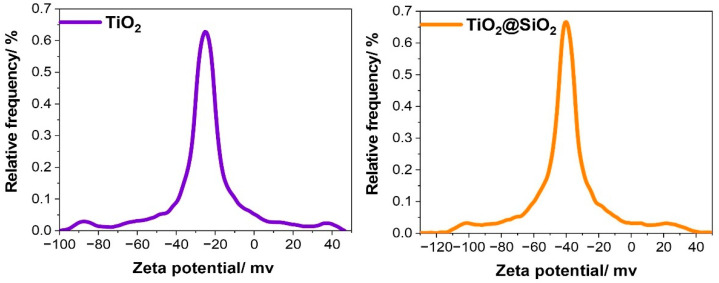
Diagrams of zeta potential for TiO_2_ and TiO_2_@SiO_2_.

In our case, the mean zeta potential for TiO_2_ is approximately −27 mV, but for TiO_2_@SiO_2_ it attains −42 mV (Figure 3). The larger negative zeta potential of TiO_2_@SiO_2_ as compared to that of the neat TiO_2_ is probably due to the high pH (around 9), which results in the formation of negatively charged sites on the silica surface, i.e., Si-O^−^ according to reaction (1) [34]. Moreover, it suggests the better stability of dispersion in an aqueous solution.

Si-OH + OH^−^ → Si-O^−^ + H_2_O (1)

### 2.2. Spectroscopic Properties

**Figure 4:**
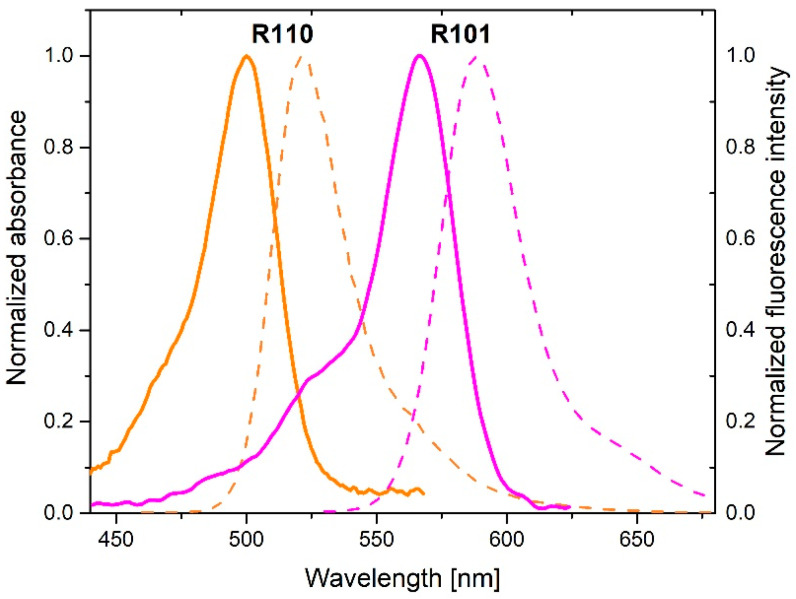
Normalized absorption (solid lines) and emission (dashed lines) spectra of donor (R110) and acceptor (R101). $\lambda_{exc}=440\;\mathrm{nm}$.

Figure 4 shows absorption and fluorescence spectra of donor (rhodamine 110 chloride) and acceptor (rhodamine 101). Strong overlap between donor fluorescence and acceptor absorption spectra as well as donor absorption and donor fluorescence spectra can be seen in the Figure 4 evidencing the possibility of FRET and energy migration, respectively. These processes can be effective on a nanoparticle if the mean distance between the interacting species is comparable or shorter than the respective critical radius. These relations depend of course on the number of donors and acceptors labelling the nanoparticle shell.

Assembly in which molecules are covalently bound to a nanoparticle in a ratio of 1 donor to 350 acceptors is labelled as D_TR_A, while a structure in which 350 donors falls on 1 acceptor is marked D_MIG_A.

**Figure 5:**
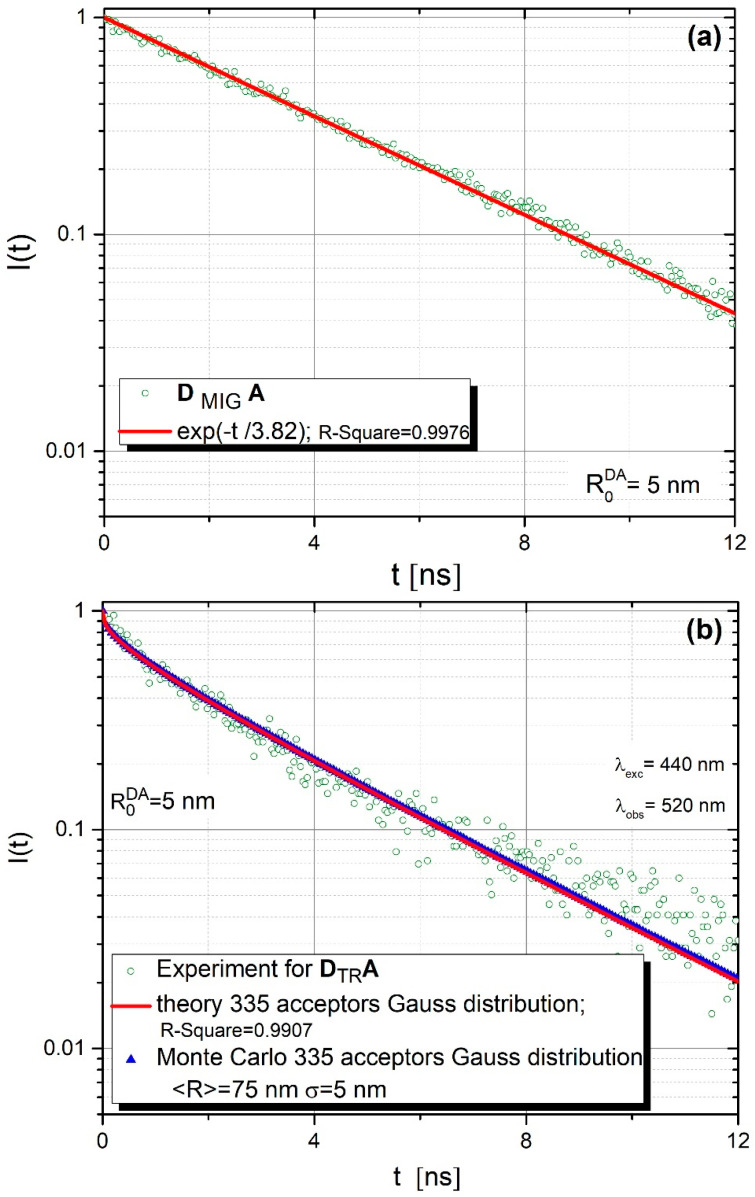
Donor fluorescence intensity decays for (**a**) D_MIG_A and (**b**) D_TR_A systems, bonded covalently to the core-shell surface.

Figure 5a,b present donor fluorescence intensity decays for D_MIG_A and D_TR_A systems, bonded covalently to the core-shell surface. Hollow green circles correspond to experimental data, blue triangles (Figure 5b) represent results of relevant Monte-Carlo simulations and a solid red line (Figure 5b) was plotted based on the theoretical predictions of the FRET model (Equation (2) in this paper).

In the case of the D_MIG_A arrangement (Figure 5a), where one deals virtually only with donors, the observed fluorescence intensity decay is practically monoexponential with the lifetime τ = 3.82 ns. This is quite typical of systems in which energy migration is a dominant or exclusive process [24,35,36,37]. The experimental data obtained remain in excellent agreement with those of Monte Carlo simulations. The best fit between experimental data and Monte Carlo simulations were obtained for *N_D_*/*N_A_* = 335/1. This value is very close to 350, resulting from the experimental procedure. Energy transfer parameters used in Monte Carlo simulations were determined from the spectroscopic measurements (absorption and fluorescence spectra, donor fluorescence lifetime) performed by us (donor fluorescence quantum yield $\eta_{0D}=0.98$,$\;\tau_{0D}$ = 3.82 ns, critical distances: $R_{0}^{DD}=46\;Å,\;R_{0}^{DA}=50\;Å$) and were fixed during the simulation process. However, for the D_TR_A structure, as revealed in Figure 5b, one observes nonexponential and generally faster fluorescence intensity decay, which is a result of efficient direct energy transfer from the donor to closely located acceptors (no energy migration).This time experimental data could be very well described not only by Monte-Carlo simulations but also by Equation (2) resulting from the analytical model for *N_D_*/*N_A_* = 1/335 [24].

Besides experimental data, Figure 5b shows the results of the theoretical model (Equation (2)) and Monte Carlo simulations. Monte Carlo simulations were carried out based on the aforementioned energy transfer parameters describing the considered donor (R110)–acceptor (R101) assembly. The Förster radius attains $R_{0}^{DA}$ = 5 nm while the average value of the orientation factor considered *κ*^2^ = 0.476 (static case), as discussed in [24]. As already mentioned, the Monte Carlo data shown in Figure 5b was obtained based on the extended model, taking into account the size variation of core-shell nanoparticles. Details regarding the application of the Gaussian function to comprise core-shell nanoparticles radii while modelling electronic excitation energy transfer are discussed elsewhere [24]. The formula expressed by Equation (2) was used to find the least squares best fit of the theoretical curve with experimental data for the donor fluorescence intensity decay. The best fit was obtained for the mean core-shell radius 〈R〉 = 75 nm and the variance σ = 5 nm for 335 attached acceptors. In view of Figure 5b, very good agreement is evident between the theoretical predictions (based on Equation (2)) and the Monte Carlo simulations for the presumed number of acceptors.

**Figure 6:**
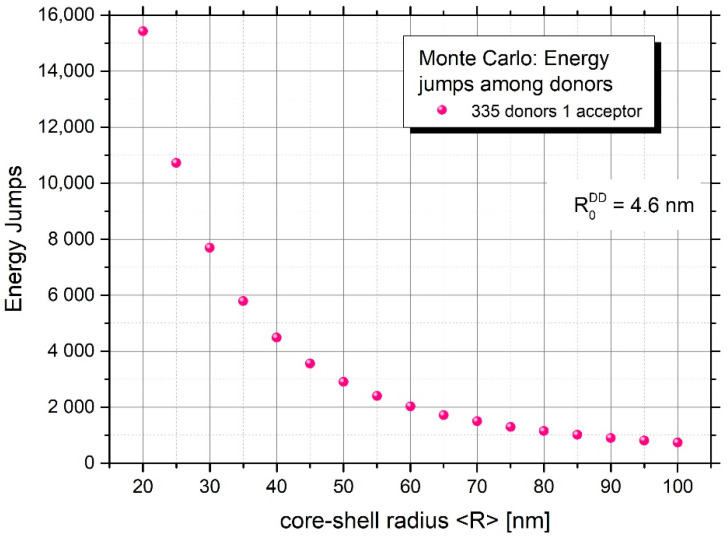
Mean number of excitation energy jumps in the presence of energy migration for nanoparticles of different mean radius.

**Figure 7:**
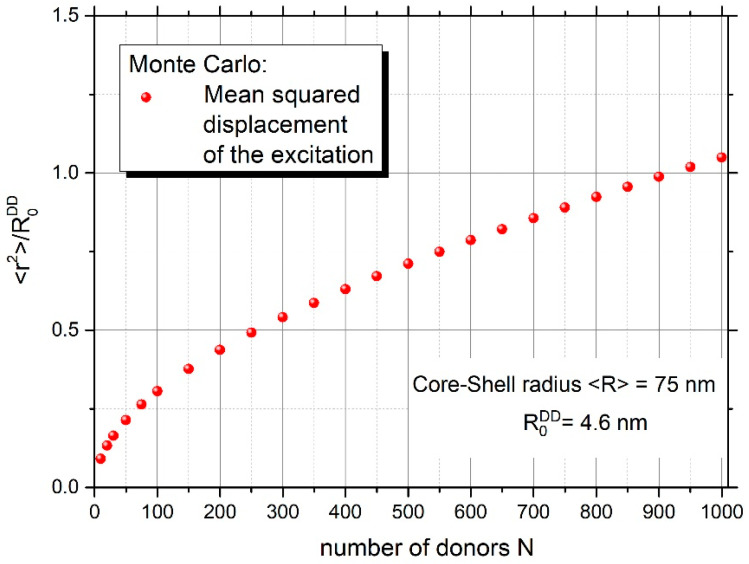
Relative mean squared displacement of excitation energy versus the number of donors on the nanoparticle.

Figure 6 and Figure 7 yield information on the mean number of excitation energy jumps and the range of energy migration, respectively. Both figures concern the case of strong energy migration (D_MIG_A). It can be seen that upon experimental conditions (〈R〉 = 75 nm) energy migration is a very intensive process and the excitation can jump many times (about 1325 times). Simultaneously, it can be seen from Figure 7 that despite a huge number of jumps, excitation energy remains localized relatively close to the initially excited site, as revealed by the value of relative mean squared displacement close to 0.6 for N = 335 donors. This is probably the result of strong remigration of excitation on a spherical nanoparticle indicating the non-Markov character of this stochastic process [38,39].

## 3. Materials and Methods

### 3.1. Materials and Reagents

All reagents and solvents were of analytical grade and used without further purification. Ammonia solutions (25%), sodium citrate dihydrate, tetraethyl orthosilicate (TEOS), 3-(aminopropyl)trimethoxysilane (APTMS), and Fmoc-glycine (Fmoc-Gly-OH) were used as a control sample; piperidine, *N*,*N*′-diisopropylcarbodiimide (DIC), 4-dimethylaminopyridine (DMAP), *N*,*N*-dimethylformamide (DMF) were purchased from Sigma-Aldrich (Poznan, Poland). All samples were prepared using deionized water (Hydrolab, Poland).

### 3.2. Synthesis

The TiO_2_@SiO_2_ and TiO_2_@SiO_2_-(CH_2_)_3_-NH_2_ structures were obtained according to the method outlined recently by Szczepańska et al. [27]. A detailed description of the syntheses is provided below.

#### 3.2.1. TiO_2_@SiO_2_

In the first stage of TiO_2_@SiO_2_ core-shell nanostructures synthesis, 50 g of titanium dioxide dissolved in the mixtures of 100 mL of ethanol and 30 mg of sodium citrate. Then, the solution was vigorously stirred, and pH was adjusted to 9 with ammonia solutions. Next, 10 mL of TEOS was added to the solution. The finished core-shell nanostructures were collected by the centrifugation and washed with ethanol several times. In the last stage, core-shell nanostructures were calcinated at high-temperature at 550 °C for 6 h due to the increase in the number of active amino groups onto the surface of core-shell nanostructures.

#### 3.2.2. TiO_2_@SiO_2_-(CH_2_)_3_-NH_2_

In the case of the synthesis of TiO_2_@SiO_2_-(CH_2_)_3_-NH_2_, 100 mg of TiO_2_@SiO_2_, 0.7 mL 3-(aminopropyl)trimethoxysilane (APTMS), and 1.5 mL of toluene were added to a round bottom flask. Next, the mixture was vigorously stirred and heated at 120 °C for 24 h. After this time, the solution was cooled, and the product was centrifuged and washed with dichloromethane and diethyl ether.

#### 3.2.3. TiO_2_@SiO_2_-(CH_2_)_3_-NH-D/A

The TiO_2_@SiO_2_-(CH_2_)_3_-NH-D/A nanocomposite was synthesized by mixing 20 mg of TiO_2_@SiO_2_-(CH_2_)_3_-NH_2_ and a 35 mg mixture of rhodamine 110 chloride and rhodamine 101 in various mass ratios (1:350 and 350:1). Next, 50 µL of DIC dissolved in about 3 mL of DMF was added. Finally, the mixture was stirred in an ice bath for 24 h, and after that the product was purified with a mixture of DMF and methanol, centrifuged, and dried. The sample was dissolved in 1 mL of methanol.

**Scheme 2:**
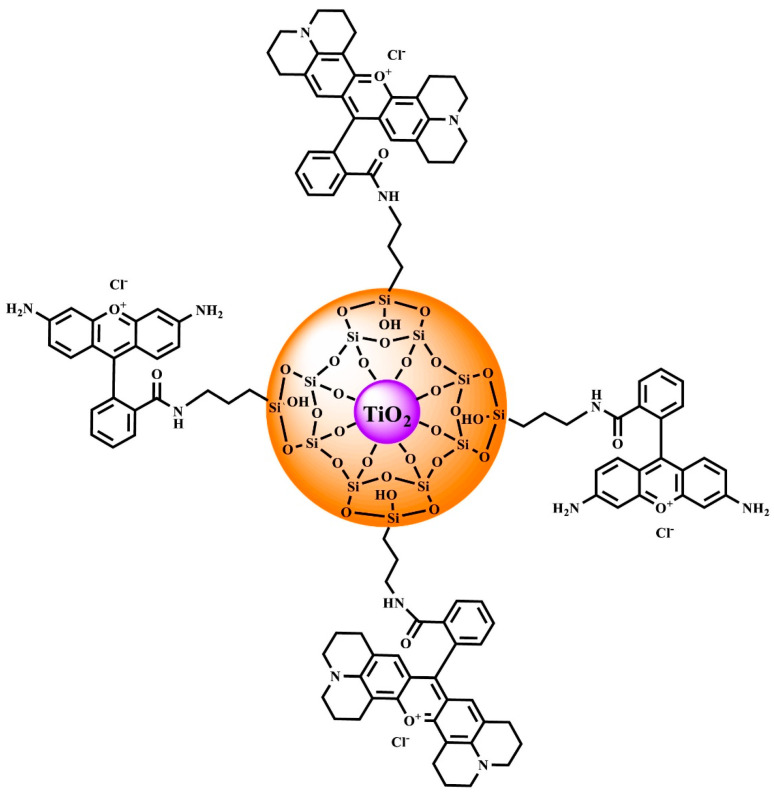
Illustrative picture of TiO_2_@SiO_2_-(CH_2_)_3_-NH-D/A.

The proposed structure of the designed nanocomposite is visualized in Scheme 2.

### 3.3. Methods

#### 3.3.1. Characteristics of Nanostructures

Transmission electron microscopy (TEM) measurements were performed with a Tecnai G2 Spirit BioTWIN by FEI (Eindhoven, The Netherlands). Samples were dispersed in ethanol and sonicated. FT-IR spectra were obtained with a Bruker IR IFS66 (Ettlingen, Germany) Fourier transform infrared spectrometer (spectral range 4000–400 cm^−1^). Samples were prepared by the standard KBr pellet method. The zeta potential was measured using the Electrophoretic Light Scattering method (ELS). Measurements were carried out on the Litesizer 500, ANTON-PAAR (Graz, Austria) for ELS > ±1000 mV. The UV-Vis spectra were taken via UV-Vis spectrophotometer with a Perkin Elmer, Lambda 650 model (Shelton, CT, USA). The measurement was carried out at 298 K using 1-cm-thick quartz spectrophotometric cuvettes in 2% (*v*/*v*) solution in DMF over the 200–800 nm range. The measurements of fluorescence spectra and fluorescence intensity decays were performed using a multifunctional pulsed spectrofluorometer constructed in our laboratory [40].

The excitation source used in our work was a Picoquant LDH440 laser diode emitting pulses at 440 nm of FWHM = 280 ps. The apparatus was configured in the front face mode. Fluorescence intensity decays were analyzed using FluoFit Pro software 4.6 (PicoQuant, Berlin, Germany).

#### 3.3.2. Theoretical Model and Monte Carlo Simulations

For a small number of donors and an arbitrary number of acceptors chemically attached to the core-shell surface (*N_D_* << *N_A_*), multistep energy migration between donors can be neglected and nonradiative energy transfer (Förster resonance energy transfer, FRET) occurs in a single step from a donor to a vicinal acceptor. Recently, we developed a FRET model dedicated to spherical core-shell nanostructures yielding the expression for the donor fluorescence intensity decay in the case of single step energy transfer [24]. Taking into account that in the majority of experimental procedures, one obtains core-shell nanoparticles characterized by a certain distribution of their radii, the following equation for the donor fluorescence decay has been obtained:

(2) $$I\left(t\right)=I_{0}\;exp\left(-\frac{t}{\tau_{0D}}\right)\;\int_{0}^{\infty }f_{G}\left(R\right){\left[\frac{1}{2}\int_{0}^{\pi }exp\left(-\frac{t}{\tau_{0D}}\;\frac{{\left(R_{0}^{DA}\right)}^{6}}{{\left[2R^{2}\left(1-cos\theta \right)\right]}^{3}}\right)\;sin\theta d\theta \right]}^{N_{A}}dR,\;$$

where

(3) $$f_{G}\left(R\right)=\frac{1}{C}\;exp\left(-\frac{{\left(R-\langle R\rangle \right)}^{2}}{2\sigma^{2}}\right)$$

is the truncated (to positive R values of core-shell radius) normal probability density function parametrized by mean value 〈R〉 and variance σ with the normalization constant C:

(4) $$C=\int_{0}^{\infty }exp\left(-\frac{{\left(R-\langle R\rangle \right)}^{2}}{2\sigma^{2}}\right)dR$$

$N_{A}$ is a number of attached acceptors, $\tau_{0D}$ is the mean fluorescence lifetime of the excited donor in the absence of acceptor and $R_{0}^{DA}$ is the critical radius for energy transfer. Mention should be made that the expression (1) holds true if no multistep energy migration between donors occurs. Such an analytical expression has not been obtained until now in the general case of energy migration and transfer on a spherical core-shell nanoparticle. Instead, the Monte-Carlo method can be applied to quantitatively analyze experimental data.

The Monte-Carlo method has been successfully applied to simulate stochastic processes (for a broad review see [41]). Mention should be made that in a Monte-Carlo simulation we can obtain not only information on the experimentally accessible observables like the donor fluorescence intensity decay, but also non-measurable quantities, for example the mean number of excitation energy jumps among molecules, the mean squared displacement of excitation energy or the local concentrations of fluorophores in various systems to characterize in depth multistep energy migration [35,42,43].

Suppose that we have a two-component system in which excitation energy can be incoherently transferred between $N_{D}$ donors $\left(D\right)$ and $N_{A}$ acceptors $\left(A\right)$ randomly distributed on the surface of a core-shell nanoparticle with the radius R. Each molecular configuration of the system is characterized by the location of molecules $r_{i}$ and orientation $\Omega_{i}$ representing the set of angular coordinates necessary to specify the orientation of the transition dipoles. In accordance with the theoretical model [24] we include herein also the probabilistic nature of core-shell nanoparticle sizes. Therefore, we assume that the radius of core-shell nanoparticlses on which molecules were uniformly distributed was drawn for each configuration of molecules from the Gauss distribution with the mean value 〈R〉 and variance σ.

The distance and dipole orientation dependent transfer rate from the *j*-th *X* molecule to the *i*-th *Y* molecule $\left(X,Y\in \left\{A,D\right\}\right)$ is denoted by $w_{x_{i}x_{j}}^{XY}$. For the Förster mechanism of resonance energy transfer, the quantity $w_{x_{i}x_{j}}^{XY}$ is given by [13].

(5) $$w_{x_{i}x_{j}}^{XY}=\frac{1}{\tau_{0X}}{\left(\frac{R_{0}^{XY}}{r_{ij}}\right)}^{6}\;$$

where $r_{ij}$ is the distance between the *i*-th and *j*-th molecule and $R_{0}^{XY}$ is the Förster radius for X→Y excitation transfer. The constants $\tau_{0D}$ and $\tau_{0A}$ are the lifetimes of donor and acceptor molecules, respectively, measured in the absence of the intermolecular energy transfer.

In this paper we used so called “step by step” method which makes use of the random-number generator for the cyclic formulation of answers to two questions: when any of the given fluorescent processes takes place and what kind of a process it is. The detailed simulation algorithms are based on the Gillespie procedure [44], further developed and adapted to describe the energy migration and transfer phenomena in disordered concentrated two component systems [35,36,45,46], uniaxially oriented polymer films [47,48], polypeptides labeled with multiple fluorophores [11,49], porous nanolayers [42,43], and core-shell spherical nanoparticles [24]. This paper closely follows the algorithm presented in [44] and modified in [24,45].

In the Monte-Carlo simulation provided herein, the donor fluorescence intensity decay is obtained by dividing the time scale into an appropriate number of identical intervals. Although this number may be arbitrarily selected it is usually taken as a multiple of 1024 in correspondence with the number of time channels used in the time correlated single photon counting measurement of the decay. In the case of this work it is equal to 4096. If a photon emission at the time $t_{i}$ is “observed”, then the number of photons is increased in the respective “channel”. Finally, the normalized decay curve (histogram) is obtained using a simple formula:

(6) $$I\left(t_{k}\right)=1-\left(\sum_{j=1}^{k}n_{j}/\sum_{j=1}^{k_{max}}n_{j}\right),\;t_{k}=\left(\frac{k}{k_{max}}\right)\;T,\;k=1,\ldots ,\;k_{max}$$

where $n_{k}$ denotes the number of photons in the *k*-th channel, $k_{max}$ is the total number of all channels, and T is the total observation time.

## 4. Conclusions

In summary, the new TiO_2_@SiO_2_-(CH_2_)_3_-NH-D/A strongly fluorescent nanocomposite was successfully fabricated in three steps. In the first step, TiO_2_-SiO_2_, core-shell nanostructures were obtained by the Ströber method. Next, functionalization with amino groups was performed. Finally, rhodamine 110 chloride as a donor and rhodamine 101 as an acceptor were attached. The nanocomposite was characterized using transmission electron microscopy and Fourier-transform infrared spectroscopy. Donor fluorescence intensity decay studies deliver more detailed information on the contribution of a single step and multistep energy transfer on a given nanoparticle. Both processes can be very accurately described by Monte-Carlo simulations or in the case of single step energy transfer also by the analytical model. The obtained results allowed us to determine the ratio of the mean number of donors to acceptors in both cases. The value obtained (*N_D_*/*N_A_* = 1/335) occurred very close to that resulting from the experimental procedure (1/350) which additionally confirms the physical sense of the performed analysis.

Important information was also derived on the range of energy migration and the number of excitation energy jumps. It was shown that the excitation energy remains quite well localized in the vicinity of the initially excited molecule (${\langle r\rangle }^{2}/R_{0}^{DD}\approx$ 0.6), despite many jumps in the donor ensemble (1325), which indicates the non-Markov character of energy transport on the spherical nanoparticle.

## Data Availability

Not applicable.
